# Lexicogrammatical profiling of ASD: cognitive-functional mapping and diagnostic implications

**DOI:** 10.3389/fnhum.2025.1704950

**Published:** 2026-01-29

**Authors:** Sumi Kato, Kazuaki Hanawa

**Affiliations:** 1 Department of Neuropsychiatry, Graduate School of Medicine, Hirosaki University, Hirosaki, Japan; 2 Faculty of Management and Law, Aomori Chuo Gakuin University, Aomori, Japan; 3 Natural Language Processing Lab, Graduate School of Information Sciences, Tohoku University, Sendai, Japan

**Keywords:** autism spectrum disorder (ASD), natural language processing (NLP), machine learning, diagnostic assessment, corpus, lexicogrammatical discriminator, systemic functional linguistics (SFL)

## Abstract

**Introduction:**

Previous corpus-based study first established an annotated dataset of autism spectrum disorder (ASD) discourse, and subsequent modeling of lexicogrammatical patterns distinguished ASD from non-ASD discourse with high performance (accuracy 80%, precision 82%, sensitivity 73%, specificity 87%). That line of research further identified 46 statistically significant discriminators, of which 20 were analyzed in detail. The present study examines 18 additional discriminators and situates them within cognitive-functional domains to clarify their diagnostic relevance. Findings refine the language–cognition interface in ASD and extend the utility of lexicogrammatical profiling for assessment.

**Methods:**

The Tag Linear Model was employed to identify lexicogrammatical features that distinguish ASD and non-ASD discourse. Logistic regression with 10,000 bootstrap iterations was applied to establish statistical significance. Although DNN models yielded higher predictive accuracy, the linear model provided transparent identification of discriminators.

**Results:**

Of the 135 items analyzed, 46 were confirmed as statistically significant discriminators (*p* < 0.05). Eighteen of these, not previously examined, were analyzed in the present study. The discriminators were mapped onto 12 cognitive-functional domains, including working memory, executive functioning, joint attention, predictive processing, and weak central coherence. The results reveal distinctive patterns across multiple domains, including reduced use of benefactive auxiliaries, relational attributive clauses, obligation modality, evaluative and gradational resources, and mimetic onomatopoeia, reflecting systematic constraints in abstraction, perspective-taking, and pragmatic orientation.

**Discussion:**

These findings demonstrate that choice patterns of lexicogrammar in ASD reflect domain-specific cognitive constraints. Interpreting the 18 discriminators within 12 cognitive-functional domains provides a linguistically grounded perspective on the neurocognitive profile of ASD and offers implications for future diagnostic and intervention research.

## 1 Introduction

Autism spectrum disorder (ASD) is a neurodevelopmental condition defined by persistent difficulties in social communication and interaction across diverse contexts, along with restricted and repetitive patterns of behavior, activities, and interests (American Psychiatric Association, 2013). A central manifestation is impairment in social communication, most commonly described as pragmatic impairment (Perkins and Firth, 1991; Martin and McDonald, 2003). Pragmatic impairment refers to difficulties in language comprehension and production at the pragmatic level, encompassing the effective use of language in social contexts. These difficulties include adjusting linguistic register to situational formality, interpreting non-literal expressions such as idioms, metaphor, irony, and sarcasm, and managing discourse in ways that support interpersonal engagement. Such impairments are distinguished from deficits in the structural aspects of language (e.g., morphology, syntax, and vocabulary), instead centering on limitations in the integration of language with social cognition.

Clinical research has converged on the view that pragmatic impairment should be analyzed comprehensively, taking into account linguistic, nonverbal, and cognitive factors. Studies have identified multiple potential sources, including neurological, cognitive, symbolic, and sensorimotor dysfunctions (Perkins, 2010; Martin and McDonald, 2003; Scobbie, 2005; Murdoch, 1990). Perkins (2010) proposed a four-domain framework for pragmatics: (i) semiotic, which covers linguistic aspects (phonology, prosody, morphology, syntax, semantics, and discourse) together with nonverbal resources such as gesture, gaze, facial expression, and posture; (ii) cognitive, which involves inferencing, theory of mind, executive function, memory, as well as emotions and attitudes; (iii) motor, which encompasses the bodily means of communication; and (iv) sensory, which concerns hearing and vision as channels for linguistic and nonverbal information. This account places cognitive dysfunction at the center of pragmatic impairment, while linguistic and sensorimotor factors are treated as secondary.

Clinical observations also report individuals with ASD who display relatively intact structural language but continue to experience substantial difficulty in effective communication. These observations underscore the critical role of cognitive capacities such as inference, executive functioning, and memory in sustaining successful interpersonal interaction. On this basis, many accounts in the clinical literature have posited a close association between cognition and pragmatic impairment (Perkins, 2010). More recent work further emphasizes this link: Schaeffer et al. (2023), for example, reviews pragmatic and structural findings together and concludes that heterogeneity in ASD language profiles can only be understood by relating linguistic behavior to cognitive functions across domains. Similarly, Frost et al. (2024) show that autistic adults’ ability to interpret indirect requests depends on their use of contextual cues, with performance predicted by inferential processing skills rather than diagnostic status.

Concrete linguistic investigations that adopt a cognitive perspective have typically concentrated on isolated grammatical domains. Examples include research on modality (Perkins and Firth, 1991; Nuyts and Roeck, 1997; Tager-Flusberg, 1997; Perkins, 1983; Kato, 2021), relative clauses (Durrleman and Delage, 2016; Durrleman et al., 2016), and syntax (Eigsti et al., 2007; Paul and Norbury, 2012; Park et al., 2012; Terzi et al., 2016; Martzoukou et al., 2017; Durrleman et al., 2015; Ambridge et al., 2020). These studies have linked specific linguistic phenomena to cognitive processes such as working memory (WM) and inferential ability. More recent contributions have extended this line of inquiry: Kalandadze et al. (2021) demonstrate that metaphor interpretation depends strongly on grammatical competence; Cano-Villagrasa et al. (2025) report that children with ASD and comorbid epilepsy show impaired auditory comprehension across multiple grammatical structures, including passives, with deficits not confined to that construction. Kissine et al. (2025), in turn, documents cases of unexpected bilingualism, demonstrating that grammatical development in autism may follow atypical yet internally coherent pathways. Despite these advances, existing studies remain confined to specific grammatical phenomena and do not amount to a comprehensive account of the relationship between cognition and lexicogrammatical patterns.

A more systematic approach to the relationship between language and cognition in ASD requires the use of spoken language corpora. Several corpora have been developed to capture autistic language, but most consist of raw transcripts without detailed linguistic annotation (Nadig and Bang, 2015; Hendriks et al., 2014; Parish-Morris et al., 2016), which limits their capacity to reveal underlying structures. For Japanese-speaking individuals with ASD, Sakishita et al. (2020) and Kato et al. (2022) constructed specialized corpora. The Sakishita corpus integrated 17 layers of phonetic annotation and was analyzed in relation to Autism Diagnostic Observation Schedule, Second Edition (ADOS-2) scores, thereby linking speech features to standardized diagnostic metrics. Kato et al. (2022), in contrast, focused on syntax and lexicogrammar by developing a systemic functional linguistics (SFL)-based annotation scheme that encompassed 159 features. Their corpus consisted of 1,187 recorded tasks from 186 autistic and 106 non-autistic participants, totaling approximately 1.07 million morphemes, providing an unprecedented level of structural detail; moreover, it is designed as a monitor corpus and is continually expanding.

In the framework of SFL, lexicogrammar refers to the integrated system of vocabulary and grammar, treated not as separate domains but as the two ends of a single continuum. Rather than analyzing lexis and syntax as distinct levels, SFL conceptualizes lexicogrammar as the stratum where grammatical structures and lexical choices jointly realize meaning. This perspective enables the analysis of language as a resource for enacting social and cognitive functions in context. Pragmatic impairment in ASD often manifests in atypical lexical selections and processing difficulties, making lexicogrammar a critical focus of investigation. Recent reviews in clinical pragmatics and applied linguistics further affirm the importance of linking grammatical form to cognitive processes (Andrés-Roqueta and Katsos, 2020).

Kato et al. (2024) applied machine learning to their annotated corpus to test whether lexicogrammatical patterns could distinguish ASD and non-ASD speakers in interview and narrative texts. Employing a text-plus-tag deep neural network (DNN) model with SFL features, they achieved 80% accuracy, 82% precision, 73% sensitivity, and 87% specificity, confirming the diagnostic potential of lexicogrammatical analysis. Importantly, the study demonstrated that neurocognitive differences in autism manifest not only in pragmatic features but also in systematic lexicogrammatical patterns.

The need for such supplementary, language-based diagnostic tools is underscored by the limitations of established assessments. The ADOS-2 is widely regarded as the current gold standard, yet the Autism Diagnostic Interview-Revised (ADI-R) is also widely used and shows strong validity (Falkmer et al., 2013). Despite these strengths in pediatric populations, the ADOS-2, in particular, struggles to differentiate autism from conditions such as ADHD, schizophrenia, and mood or anxiety disorders, and its structured tasks are vulnerable to masking and compensation strategies (Barlati et al., 2019; De Crescenzo et al., 2019; Gould and Ashton-Smith, 2011; Hull et al., 2017; Lai and Baron-Cohen, 2015). Adult diagnosis is further complicated by the absence of caregiver developmental histories and the limited reliability of self-report (Frith, 2003; Berthoz and Hill, 2005). These factors reduce the effectiveness of current gold standards for adults and highlight the need for approaches that can detect systematic linguistic markers within language use itself.

Kato and Hanawa (2025) used the Tag Linear Model with logistic regression to identify discriminators distinguishing ASD from non-ASD speech. Out of 135 features, 46 emerged as significant, and 20 of these were analyzed in relation to cognitive-functional domains such as WM, inferential reasoning, joint attention (JA), weak central coherence (WCC), and agency. The present study focuses on these cognitive-functional domains, which are here treated as analytic constructs linking linguistic form to underlying cognitive processes rather than as isolated neuropsychological measures. These findings indicated that lexicogrammatical variation reflects domain-specific cognitive constraints.

The present study addresses 18 additional discriminators, with the aim of providing a more comprehensive profile of lexicogrammatical variation in ASD. By situating these features within cognitive-functional domains, the study seeks to clarify how structural language choices index underlying neurocognitive orientations. In doing so, it complements earlier analyses and contributes to a fuller and more interpretable account of the language–cognition interface in autism.

Such systematic investigation remains rare. While pragmatic and structural differences in autistic language have been widely studied, few works have examined lexicogrammar across multiple domains in direct relation to cognition. The contribution of the present study is therefore threefold: first, to advance theoretical understanding of language as a meaning-making system shaped by neurocognitive orientation; second, to reinforce the diagnostic potential of SFL-based lexicogrammatical profiling as a supplement to existing tools, in adult and adolescent assessment where conventional instruments such as the ADOS-2 and ADI-R are less reliable; and third, to support the development of language education interventions informed by lexicogrammatical findings.

## 2 Methods

This study followed the same overall design and analytic procedures as Kato and Hanawa (2025), which established and validated the Tag Linear Model for comparing proportional frequencies of lexicogrammatical features between ASD and non-ASD groups. To maintain methodological continuity while allowing independent interpretation, we provide here a concise overview of the participant groups, corpus composition, annotation scheme, and statistical analysis. Full procedural details, including feature definitions and model specifications, are available in Kato and Hanawa (2025).

### 2.1 Participants

The dataset comprised the same groups analyzed in Kato and Hanawa (2025). In total, there were 64 adults with ASD (M = 18 years, SD = 3.48) and 71 adults without ASD (M = 19 years, SD = 2.77), all native speakers of Japanese aged 14 and above. ASD participants were diagnosed by licensed clinicians according to DSM-5 criteria, primarily using the ADOS-2, with supporting assessments including the social responsiveness scale, second edition (SRS-2), WISC-IV/WAIS-III/IV, Vineland-II, autism-spectrum quotient (AQ), and PARS-TR. The non-ASD group included 17 clinically assessed neurotypical adults and 54 university students, both confirmed as non-spectrum through ADOS-2 scores. Some ASD participants presented comorbidities, reflecting the clinical heterogeneity typically observed in ASD. Further demographic details are reported in Kato and Hanawa (2025) and Supplementary Table S1. The spoken data collected from these participants constitute the corpus analyzed in the present study, as described in Section 2.2.

### 2.2 Corpus data

The corpus was identical to that described in Kato and Hanawa (2025). Semi-structured interviews were conducted in a clinical context, drawing on the ADOS-2 framework with supplementary open-ended prompts to elicit extended discourse (see Supplementary material S1). Each session was recorded, transcribed according to orthographic conventions, and segmented into clauses for analysis. Transcriptions were produced by trained research assistants using standard Japanese orthographic conventions, following the procedures described in Kato and Hanawa (2025). Only participants’ responses were analyzed; examiner prompts were excluded from the dataset.

### 2.3 Annotation scheme

All transcripts were annotated according to the SFL tagging scheme established in Kato et al. (2022). The scheme covered major lexicogrammatical features across SFL-based metafunctions. Coding reliability was monitored through multiple annotators and consensus resolution (see Supplementary Table S2).

### 2.4 Statistical analysis

The analysis followed the same overall procedure as Kato and Hanawa (2025), which established and validated the Tag Linear Model for comparing proportional frequencies of lexicogrammatical features between ASD and non-ASD groups. As demonstrated in Kato et al. (2024), deep-learning architectures such as Text + Tag DNN achieved higher predictive accuracy (80% accuracy, 82% precision, 73% sensitivity, 87% specificity) but did not allow direct identification of individual discriminators, since feature interactions were non-transparent. By contrast, the Tag Linear Model permits coefficient-based estimation of each lexicogrammatical feature’s contribution, making it suitable for the present study’s goal of clarifying how linguistic resources map onto cognitive-functional domains. Logistic regression with 10,000 bootstrap iterations was applied to estimate coefficients and confidence intervals. This procedure ensures stable parameter estimation even under high feature dimensionality. While the computational framework remains unchanged from the previous study, the current analysis extends the model to 18 additional discriminators covering 12 cognitive-functional domains. The choice of a linear model thus reflects a deliberate emphasis on interpretability and theoretical transparency.

## 3 Results

Logistic regression analysis with bootstrap resampling identified 46 lexicogrammatical items as statistically significant discriminators (*p* < 0.05) between ASD and non-ASD speakers. In Kato and Hanawa (2025), 20 of these features were analyzed, focusing on variation at the clause and phrase level. Current study addresses an additional 18 items, which likewise exhibit clear lexicogrammatical patterning relevant to ASD-related language use.

Of the 46 statistically significant features identified, the remaining 8 were excluded from interpretation due to empirical limitations or interpretive ambiguity. Constructions involving explanatory mood (*noda*) combined with negotiating particles, as well as fillers like *unto* and *kono*, reached statistical thresholds but lacked analytic tractability: *noda* did not emerge as an independent discriminator, the negotiating particles have been addressed in prior work (Kato et al., 2022; Kato and Hanawa, 2025), and the fillers were judged to reflect speaker-specific or interactional habits rather than systematic cognitive-functional patterns. Their omission reflects a conservative stance prioritizing clarity and replicability, though future research with multimodal or expanded corpora may clarify their contribution to ASD-related language use.

**Figure 1:**
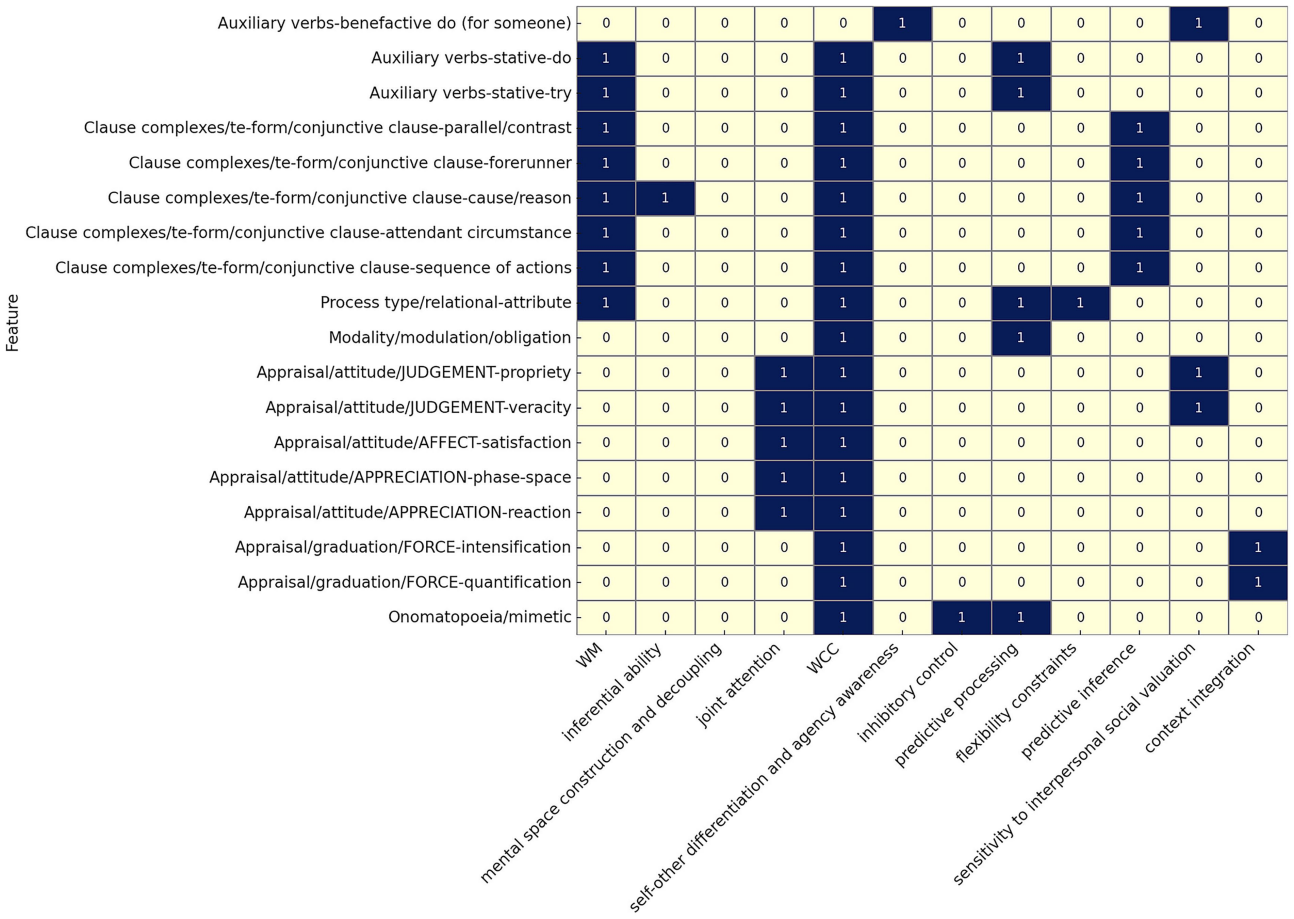
Syntactic variation in ASD: lexicogrammatical discriminators and cognitive implications.

Table 1 summarizes the 18 discriminators examined here. Figure 1 provides a functional categorization, mapping each discriminator to the cognitive domains it most clearly reflects. Table 1 and Figure 1 serve as the empirical basis for the interpretive framework developed in the Discussion. The 18 discriminators examined in this study are briefly summarized below; fuller interpretations are provided in the Discussion.

**Table 1:** Statistical significance of lexicogrammatical discriminators in differentiating ASD from non-ASD lexicogrammatical choices.

Lexicogrammar	Mean	SD	*p*-value
Auxiliary verbs-benefactive do (for someone)	−0.0052	0.0018	0.0031
Auxiliary verbs-stative-do	−0.0183	0.0075	0.0148
Auxiliary verbs-stative-try	−0.007	0.0032	0.0256
Clause complexes/*te*-form/conjunctive clause-parallel/contrast	−0.0247	0.0069	0.0004
Clause complexes/*te*-form/conjunctive clause-forerunner	−0.0074	0.003	0.0136
Clause complexes*/te*-form/conjunctive clause-cause/reason	−0.0411	0.0103	0.0001
Clause complexes/*te*-form/conjunctive clause-attendant circumstance	−0.0185	0.0079	0.0181
Clause complexes/*te*-form/conjunctive clause-sequence of actions	−0.0223	0.0084	0.0079
Process type/relational-attribute	−0.0849	0.0316	0.0072
Modality/modulation/obligation	−0.0042	0.0015	0.0047
Appraisal/attitude/JUDGMENT-propriety	−0.0096	0.0042	0.0235
Appraisal/attitude/JUDGMENT-veracity	−0.0124	0.0052	0.0163
Appraisal/attitude/AFFECT-satisfaction	−0.0328	0.0049	0
Appraisal/attitude/APPRECIATION-phase-space	−0.0024	0.0011	0.0236
Appraisal/attitude/APPRECIATION-reaction	−0.0533	0.0206	0.0098
Appraisal/graduation/FORCE-intensification	−0.1595	0.021	0
Appraisal/graduation/FORCE-quantification	−0.0122	0.0057	0.0333
Onomatopoeia/mimetic	−0.0126	0.0059	0.0315

(i) Auxiliary verbs – benefactive do (-*te ageru*)

Significantly underused in ASD discourse, suggesting difficulties with marking intentional actions as benefiting others, linked to self–other differentiation and agency representation.

(ii) Auxiliary verbs – *-teshimau* (end up doing) and *-temiru* (try doing)

Significantly less frequent in ASD discourse, reflecting difficulties in integrating intention, outcome, and evaluative or exploratory stance, linked to WCC, executive function constraints, and predictive processing anomalies.

(iii) Clause complexes/*te*-form

Significantly less frequent in ASD discourse, reflecting difficulties with discourse-level integration and contextual inference.

(iv) Process type – relational attributive

Significantly less frequent in ASD discourse, suggesting constraints in abstraction and generalization linked to executive functioning and predictive processing.

(v) Modality – modulation/obligation

Significantly less frequent in ASD discourse, reflecting predictive processing anomalies, local-detail bias, and difficulties in adopting normative frameworks for social negotiation.

(vi) Appraisal/attitude (judgment, affect, appreciation) + graduation/force (intensification, quantification)

Judgment—propriety and veracity, Affect—satisfaction, and Appreciation—reaction and scalarity–space were significantly less frequent in ASD discourse. Force (intensifiers, quantifiers) was also reduced, reflecting constraints in affordance perception and social learning (JA), local-detail bias (WCC), and predictive processing.

(vii) Onomatopoeia – mimetic

Significantly less frequent in ASD discourse, with selective reduction in mimetic forms, reflecting demands on predictive processing, executive functions, and context-sensitive abstraction.

## 4 Discussion

This section interprets the 18 lexicogrammatical discriminators identified in the Results in terms of underlying cognitive domains associated with ASD, including WM, executive functioning, JA, predictive processing, and WCC. Taken together with the discriminators reported in Kato and Hanawa (2025)—comprising both reduced and increased uses of specific structures—the present analysis advances a broader account of how choice patterns of lexicogrammar in ASD can be traced to domain-specific processing characteristics. The 18 discriminators are interpreted within 12 cognitive-functional domains (Figure 1), allowing syntactic variation to be related to broader neurocognitive mechanisms.

### 4.1 Benefactive perspective-taking: from implicit self to agentive other

In Japanese, certain grammatical resources explicitly require the speaker to adopt a relational perspective, encode interpersonal dynamics, and differentiate self from other. Chief among these are benefactive and recipient auxiliary verbs, which attach to the *-te* form of a main verb to express whether an action is performed for another’s benefit, received from another, or directed toward the speaker. Unlike main predicates, these auxiliaries modify the interpersonal interpretation of an event, foregrounding the alignment of agent, recipient, and speaker. Verbs such as *ageru*, *kureru*, and *morau* thus operate dually: as lexical verbs of giving and receiving, and as auxiliaries that encode benefaction, social deixis, relative status, and empathic alignment. For example, *jitensha-o naoshite-**ageru*** (I fix the bicycle for [someone]) signals action for another’s benefit, whereas *naoshite-morau* (I have someone fix [it] for me) encodes the speaker as beneficiary. The principal forms of benefactive and recipient auxiliary verbs can be illustrated as follows (Table 2):

**(i) *ageru*/*sashiageru***
  *ageru* is the neutral form. *Sashiageru* is the honorific form used when the beneficiary is of higher social status. Both indicate that the speaker or a speaker-aligned subject performs an action for the benefit of another. A full list of grammatical abbreviations used in the glosses is provided in Supplementary Table S3.
  (1) *-te ageru* (neutral)
*Watashi-wa tomodachi-ni okane-o kashite-ageta.*
  I-NOM friend-DAT money-ACC lend-TE-BEN.NEUT.PST
  (I lent money to my friend for their benefit).
  (2) *-te sashiageru* (honorific)
*Watashi-wa sensei-ni omiyage-o motte-itte-sashiageta.*
  I-NOM teacher-DAT souvenir-ACC bring-go-TE-BEN.HON.PST
  (I took a souvenir to my teacher).
**(ii) *yaru***
  *yaru* is the neutral form with a rougher or socially lower nuance. It indicates that the speaker or subject performs an action for another.
  (3) *-te yaru* (neutral)
*Watashi-wa imōto-o kuruma-ni nosete-yatta.*
  I-NOM younger.sister-ACC car-LOC ride-TE-BEN.NEUT.PST (low)
  (I gave my younger sister a ride in the car).
**(iii) *morau*/*itadaku***
  *morau* is the neutral form used when the speaker or subject receives the benefit of an action performed by another. *Itadaku* is the honorific counterpart used when the benefactor is of higher status.
  (4) *-te morau* (neutral)
*Watashi-wa haha-ni bentō-o tsukutte-moratta.*
  I-NOM mother-DAT bento-ACC make-TE-BEN.REC.NEUT.PST
  (I had my mother make a bento lunch for me).
  (5) *-te itadaku* (honorific)
*Watashi-wa sensei-ni shidō-o shite-itadaita.*
  I-NOM teacher-DAT guidance-ACC do-TE-BEN.REC.HUM.PST
  (I received guidance from the teacher).
**(iv) *kureru*/*kudasaru***
  *kureru* is the neutral form used when another person performs an action for the speaker. *Kudasaru* is the honorific form used when the benefactor is socially superior.
  (6) *-te kureru* (neutral)
*Tomodachi-ga watashi-o eki-made okutte-kureta.*
  friend-NOM I-ACC station-until send-TE-BEN.NEUT(to. speaker).PST
  (My friend gave me a ride to the station).
  (7) *-te kudasaru* (honorific)
*Sensei-ga watashi-ni shidō-o shite-kudasatta.*
  teacher-NOM I-DAT guidance-ACC do-TE-BEN.HON.PST
  (The teacher gave me guidance).

The choice of benefactive and recipient auxiliaries depends on both the direction of benefit and the speaker’s alignment with participants. These verbs add an interpersonal layer by encoding who benefits and how the speaker positions themselves socially and psychologically. Because their use requires clear self–other distinction, stable agency attribution, and flexible perspective-taking, usage patterns serve as sensitive indicators of interpersonal cognition. Differences in deployment thus reveal core dimensions of self–other representation, agency, and social cognition, especially in populations such as individuals with ASD where these domains are often atypical. The cognitive mechanisms underlying this reduced use are examined below.

**(i) Impairments in self–other differentiation**

Using *ageru* requires distinguishing self from other, with the speaker positioning themselves (or their side) as the agent acting intentionally for another’s benefit. Yet, individuals with ASD often show persistent difficulties in self–other differentiation. The interpersonal self that supports flexible perspective-taking remains underdeveloped, leaving them anchored in the ecological self—defined by direct environmental interaction without dynamic role awareness (Kato, 2024). Lacking this interpersonal frame, it is cognitively unnatural to mark actions as benefiting others, making the shift from *I act* to *I act for you* difficult. Consequently, in spontaneous discourse, ASD speakers are less likely to select benefactive forms like *ageru*.

**(ii) Impairments in sense of agency**

The use of *ageru* presupposes a stable sense of agency, requiring the speaker to recognize themselves as the intentional initiator of an action that benefits another. Individuals with ASD often show atypicalities in agency representation (Kato, 2024). While basic motor initiation may be intact, extending agency into the social domain—seeing oneself as the cause of another’s altered state or benefit—is markedly weakened. Linguistically, this results in a reduced tendency to frame actions as socially meaningful interventions. Instead of adopting the agentive stance of giving, ASD speakers may describe only the physical occurrence of events, leaving constructions that demand agentive, benefactive framing, such as *ageru*, less accessible in spontaneous discourse.

**Table 2:** Benefactive and recipient auxiliary verbs in Japanese: meanings, directionality, and social register.

Verb	Basic meaning	Direction of benefit	Social role/Register	Corresponding honorific/Humble form
*ageru*	To give (do something for another)	Speaker/subject → other	Neutral/standard	*sashiageru* (honorific)
*yaru*	To give (do something for another)	Speaker/subject → other	Informal / used toward animals or lower status	—
*morau*	To receive (benefit from another’s action)	Other → speaker/subject	Neutral	*itadaku* (humble)
*kureru*	To give (another does something for the speaker)	Other → speaker	Neutral	*kudasaru* (honorific)

**(iii) Difficulty in simulating the recipient’s perspective**

Using *ageru* requires not only self-agency but also the ability to simulate the recipient’s viewpoint—to recognize the act as beneficial from their perspective. This involves shifting from one’s own frame of reference to the imagined experience of the other. Individuals with ASD struggle to flexibly adopt others’ perspectives, both perceptually and socially. Without spontaneously simulating how an action affects another, the evaluation *this is a favor for you* does not arise, weakening the motivation to choose benefactive forms. As a result, actions are more often expressed from neutral or self-centered standpoints, and auxiliaries encoding an empathetic benefactor stance, such as *ageru*, are systematically underused.

**(iv) Weakened sensitivity to interpersonal social valuation**

Using *ageru* requires recognizing that an act is socially framed as a favor, not merely as a physical event. This presupposes evaluating actions within an interpersonal value system, where certain behaviors directed toward others imply assistance, generosity, or support. For individuals with ASD, such social-affective dimensions are less salient, and actions tend to be encoded in terms of physical causality rather than embedded in culturally mediated value structures. Consequently, they may not conceptualize their own acts as favors but simply as events. Without this layer of valuation, benefactive auxiliaries like *ageru*, which presuppose recognition of positive interpersonal impact, become less cognitively motivated, reflecting a broader orientation toward physical rather than social-affective experience.

In sum, the reduced use of *ageru* indicates difficulties with intentional benefaction and self–other differentiation. Other benefactive forms showed no group differences, likely due to the topic-restricted interview setting, which constrained the pragmatic use of honorific auxiliaries (e.g., *sashiageru*, *itadaku*, *kudasaru*) and informal forms like *yaru*. Broader interactional settings would be needed to assess these verbs’ distribution more fully.

### 4.2 Cognitive constraints on -*teshimau* and -*temiru*

The Japanese auxiliary verbs -*teshimau* and -*temiru* go beyond simple event descriptions by linguistically incorporating intention, outcome, evaluation, and an exploratory stance. These forms function as lexicogrammatical resources that reflect complex cognitive operations.

-*teshimau* is multifunctional, with at least four distinct uses: aspectual completion, regret, unintended result, and impulsive completion. Representative examples are given below:

(8) *Sono hon-o yonde-shimatta*
  that book-ACC read-TE-shimau-COMP-PST
  (I ended up finishing reading that book).
  (9) *Shukudai-o wasurete-shimatta*
  homework-ACC forget-TE-shimau-COMP-PST
  (I ended up forgetting my homework [accompanied by regret]).
  (10) *Densha-de nete-shimatta*
  train-LOC sleep-TE-shimau-COMP-PST
  (I ended up falling asleep on the train [unintended result]).
  (11) *Tsui donatte-shimatta*
  unintentionally shout-TE-shimau-COMP-PST
  (I ended up shouting [impulsive completion]).
  In contrast, -*temiru* consistently frames the action as a trial attempt, expressing an exploratory attitude that accepts uncertain outcomes. It signals the speaker’s intention to *try* something and observe the result. An example is shown below:
  (12) *Kare-ni tazunete-mita*
  he-DAT ask-TE-miru-TRY-PST
  (I tried asking him [trial, accepting uncertain result]).

The reduced use of *-teshimau* and *-temiru* in ASD discourse reflects interrelated cognitive constraints. These constraints are examined below in terms of WCC, executive functioning, and predictive processing.

**(i) WCC and local processing bias**

WCC, characteristic of ASD, prioritizes local detail over global integration. Evaluative forms such as (9) compress intention and outcome into a unified evaluative package. Yet in ASD, speakers often prefer plain descriptive clauses like *Shukudai-o wasureta* (I forgot the homework), omitting evaluative stance. Similarly, (12) is replaced by *Kare-ni tazuneta* (I asked him), which reports the action without encoding uncertainty. The reduced frequency of these auxiliary verbs thus reflects a cognitive style favoring discrete, literal observations over integrated, evaluative constructions in ASD.

**(ii) Executive function constraints**

The evaluative uses of *-teshimau* demand simultaneous representation of intention, outcome, and stance in WM. For example, (10) encodes the plan not to fall asleep, the unintended outcome of falling asleep, and the evaluative implication of error. Cognitive flexibility is likewise required, as in (11), where a simple act of shouting is reclassified as an impulsive lapse. *-temiru* requires similar resources: flexibility to reframe an action as an experiment and relational memory to consolidate patterns of exploratory behavior across experiences. Research has shown limitations in these executive domains in ASD, explaining the rarity and reduced naturalness of these auxiliaries.

**(iii) Predictive processing anomalies**

Both auxiliaries depend on predictive modeling. *-teshimau* compresses the mismatch between expected and actual outcomes, as in (9). Such usage requires detecting prediction error and attaching evaluative significance. *-temiru* presupposes tolerance of indeterminacy, framing actions as open-ended trials with uncertain futures, as in (12). In ASD, predictive mechanisms often overemphasize immediate sensory input while underweighting prior expectations, making both error-based evaluation and uncertainty tolerance less accessible (Van de Cruys et al., 2014). As a result, auxiliaries are replaced by plain, episodic descriptions that minimize abstraction.

In sum, the reduced use of *-teshimau* and *-temiru* in ASD discourse reflects interrelated cognitive constraints: a detail-focused style associated with WCC, limitations in executive resources such as WM and cognitive flexibility, and predictive anomalies that impair error monitoring and uncertainty management. These auxiliaries function as gateways between concrete action and higher-order meaning-making, but in ASD they are displaced by literal, episodic reporting such as *I forgot the homework* or *I asked him*, instead of using these auxiliaries.

### 4.3 The *te*-form as an implicit and polyfunctional construction

Kato and Hanawa (2025) discussed inferential difficulties in ASD. One structure that exemplifies these challenges is the Japanese *te-form* when used to express cause or reason. Unlike explicit causal connectives such as *kara* or *node* (because, since), the *te-form* functions as a conjunctive device that links two clauses without overtly marking the causal relation. This implicitness requires the hearer to infer the causal link from context, thereby placing greater demands on integrative processing.

Consider the following example:
  (13) *Okane-ga naku-**te**, kuruma-ga kae-nakatta*
  money-NOM not.exist-GER car-NOM buy-NEG-PAST
  (I did not have money, so I could not buy it).
  Contrast this with an explicit causal construction:
  (14) *Okane-ga naka-tta **kara**, kuruma-ga kae-nakatta*
  money-NOM not.exist-PAST because car-NOM buy-NEG-PAST
  (Because I did not have money, I could not buy it).

While the two sentences are semantically comparable, the former lacks an explicit causal marker. In (13), the causal link must be inferred from context, whereas (14) provides overt syntactic encoding. For individuals with ASD, who often exhibit difficulties with inference, contextual integration, and cognitive flexibility, such implicitness imposes considerable processing demands. The core problem lies in the *te-form’s* polyfunctionality. Despite being a single morpheme, *te* is used to express a wide range of interclausal semantic relations, including cause or reason, temporal succession, contrast, forewarning, and attendant circumstance (Nihon-Kijutsubunpo-Kenkyukai, 2008). Its meaning must be pragmatically inferred from context rather than being explicitly marked in syntax. The following examples illustrate this multifunctionality:

**(i) Contrastive relation**
  (15) *Kono heya-wa akaruku-**te**, tonari-no heya-wa kurai*
  this room-TOP bright-GER next-GEN room-TOP dark
  (This room is bright, and the next room is dark).
  Here the contrast emerges implicitly through semantic opposition, not through an overt adversative marker.
**(ii) Forewarning or pre-emptive framing**
  (16) *Mondai-ga hitotsu at-**te**, chichi-wa eigo-ga hanase-nai no-da*
  problem-NOM one exist-GER father-TOP English-NOM speak.can-NEG EXPL-COP
  (There’s one problem: my father cannot speak English).
  The *te-form* here introduces a general statement that sets up the main clause.
**(iii) Sequential relation**
  (17) *Te-o arat-**te**, oyatsu-o tabeta*
  hand-ACC wash-GER snack-ACC eat-PAST
  (I washed my hands and ate a snack).
  The first action frames the second chronologically.
**(iv) Attendant circumstance**
  (18) *Warat-**te** kamera-o mita*
  laugh-GER camera-ACC look-PAST
  (She looked at the camera, smiling).

The subordinate clause adds circumstantial color to the main event.

These diverse uses demonstrate the semantic flexibility of the *te-form*, which contributes to its interpretive complexity. Because the construction does not explicitly mark interclausal relations, comprehension depends on context, shared knowledge, and discourse-level cues. For neurotypical speakers, this allows flexible meaning construction; for individuals with ASD, however, it imposes substantial inferential demands. In Kato and Hanawa (2025), four subtypes of the *te-form*—contrastive relation, forewarning, sequential relation, and attendant circumstance—were statistically identified as discriminators between ASD and non-ASD groups. This finding is theoretically consistent with the high inferential and contextual-reasoning demands required to interpret these implicit constructions. Notably, two additional subtypes—adversative and resultative conditional relations—were included in the annotation scheme but did not show significant group differences in the bootstrap-based logistic regression. Examples are given below.

**Adversative relation**
  (19) *Shitte-i-**te** tasukena-katta*
  know-PROG-GER help-NEG-PAST
  (He knew, but did not help).
  The first clause sets up an expectation that is contradicted in the second.
**Resultative conditional relation**
  (20) *Kanojo-o ire-**te** juu-nin ni nat-ta*
  she-ACC include-GER ten-CL become-PAST
  (Including her, there were ten people).

The first clause functions as a condition, yielding the numerical result expressed in the second.

This lack of significance is likely due to their low frequency in the interview data rather than to any lack of inferential importance. Given their cognitive complexity, these subtypes may still serve as meaningful indicators of ASD-related processing differences and warrant further investigation in more contextually diverse environments.

The ability to resolve ambiguities among these functions hinges on discourse-level integration, a capacity often impaired in ASD due to WCC and executive function deficits in WM, flexibility and inhibitory control. Individuals with ASD tend to rely more heavily on explicit syntactic cues, and thus struggle with constructions where meaning must be inferred from pragmatic or contextual signals (Jolliffe and Baron-Cohen, 1999). In this respect, explicit connectives such as *kara*, *node*, *ba*, or *nara* may serve a compensatory function by reducing inferential demands and facilitating comprehension. These connectives make interclausal relations overt, thereby minimizing the need for higher-order inferential reasoning and enabling more reliable processing of intended meanings.

In sum, the polyfunctional and implicit nature of the *te-form* makes it a demanding construction for individuals with ASD. Its interpretation depends on contextual reasoning, which is often compromised, leading to reduced use of the *te-form* in ASD discourse. That several *te*-form subtypes emerged as discriminators indicates that implicit connective structures are especially revealing of the cognitive-linguistic processing style characteristic of ASD.

### 4.4 Relational attributive: cognitive constraints on generalization and abstraction

**Relational processes–attribution**

In the SFL framework, processes constitute the central system for construing experience through the transitivity system. The clause serves as the primary unit, and human experiences are categorized into several major Process types (see Supplementary Figure S1). Among these, relational processes construe relationships and states of being. They are typically divided into attributive, which assign qualities or classifications to a participant (Carrier), and identifying, which establish identity or equivalence between two entities. A fuller overview of the process system is provided in Kato and Hanawa (2025).

**Attributive relational process (assigning qualities or attributes)**
  (21) *Kono hon-wa omoshiroi*
  this book-TOP interesting-AST
  (This book is interesting).
**Identifying relational process (establishing identity or equivalence)**
  (22) *Kare-ga hannin da*
  he-NOM culprit be-AST
  (He is the culprit).

In ASD discourse, a marked reduction appears in the use of attributive relational clauses. This subtype assigns qualities or classifications to a Carrier—for example, in *Kono hon-wa omoshiroi* (this book is interesting), *omoshiroi* functions as the Attribute assigned to *kono hon*. Such clauses compress multiple observations into generalized characterizations, as in *Kare-wa kimae-ga yoi* (he is generous). Their reduced use in ASD reflects constraints in abstraction and generalization, consistent with WCC, executive function limitations, and predictive processing difficulties. The cognitive underpinnings of this reduction are explored in the following discussion.

**(i) WCC and local processing bias**

Relational attributive processes require synthesizing multiple observations into a generalized property attributed to a participant. For example, describing a person as generous reflects integration across diverse behavioral instances. This operation demands global meaning construction, subordinating local details to higher-order abstraction. Individuals with ASD, however, show WCC, which biases them toward local, context-independent details rather than integrated wholes (Frith, 1989; Happé and Frith, 2006).

Under WCC, attention gravitates to isolated facts and specific appearances, with less spontaneous linking into broader generalizations. Consequently, relational attributive clauses are less frequent in ASD language, with preference for descriptive clauses about concrete acts (e.g., she helped me carry my bag yesterday) rather than abstract attributions (e.g., she is kind). Labeling someone as trustworthy, for instance, presupposes noticing stability across acts such as returning items or honoring commitments. In ASD, each situation tends to be processed as a separate episode, limiting generalized attributions and leading to discourse focused on concrete, episodic observations. This pattern is consistent with evidence of detail-focused style (Happé and Frith, 2006) and atypical predictive processing (Van de Cruys et al., 2014).

**(ii) Executive function (WM, flexibility, long-term memory (LTM))**

Relational attributive processes require generalizing across behavioral instances to assign a stable property to a participant. This operation engages WM for retaining and organizing multiple observations and cognitive flexibility for shifting attention between features, while LTM supports the consolidation of recurring patterns (Miyake et al., 2000; Conway et al., 2003). WM supports attributions such as *he is reliable* by holding events like arriving on time or completing tasks, while cognitive flexibility enables grouping diverse acts under categories such as kindness. LTM supports the consolidation of general patterns and themes across episodes. Research indicates that individuals with ASD have difficulty retaining thematic or gist-based information over longer delays (Williams et al., 2017), and show selective deficits in relational memory—the ability to bind relations between elements—even when item memory remains intact (Minor et al., 2023). These limitations in abstraction may lead to discourse that favors concrete, episodic descriptions over generalized characterizations.

**(iii) Predictive processing difficulties and generalization deficits**

Relational attributive processes require abstracting over experiences to project stable properties beyond immediate events. This depends on predictive modeling, whereby prior information guides generalization and expectation. In ASD, predictive anomalies disrupt this mechanism, resulting in language that reports concrete phenomena rather than generalized attributions.

Predictive processing accounts view cognition as anticipatory: the brain builds models from prior experiences, predicts incoming data, and updates through prediction error (Friston, 2010). Relational attributions such as *he is dependable* rely on generalizing across past interactions to predict future behavior. In ASD, however, each event tends to be processed as novel, making dispositional attributions cognitively unnatural. Studies suggest that predictive mechanisms are underweighted (Pellicano and Burr, 2012) or overly precise at lower sensory levels but fail to generalize flexibly at higher levels (Van de Cruys et al., 2014).

Consequently, ASD discourse emphasizes specific events (e.g., *he waited for the bus without complaining today*) rather than generalized properties (*he is patient*). Reduced predictive generalization also impedes recognizing consistency across variable acts—such as carrying groceries or giving directions—as instances of helpfulness. Language thus privileges accuracy and specificity over abstraction, aligning with broader findings of detail-focused communication in ASD (Eigsti et al., 2011; Durrleman et al., 2022). Because relational attribution depends on integrating, predicting, and generalizing across events, predictive anomalies naturally reduce its frequency and naturalness. Instead of encoding enduring properties, ASD discourse reflects discrete, situation-bound observations.

In sum, the reduced use of relational attributive processes in ASD reflects constraints on abstraction, generalization, and flexible categorization. Within the SFL framework, these clauses require integrating diverse observations into stable attributions, yet ASD discourse favors detailed, context-bound event descriptions over generalized characterizations, consistent with local-detail processing and reduced predictive generalization.

### 4.5 Obligation modulation as a discriminator of interpersonal negotiation

In the SFL framework, the modality system is divided into two major types: **modalization** and **modulation**. Modalization covers assessments of probability (e.g., *He may be there*) and usuality (e.g., *She often goes*), whereas modulation concerns proposals rather than propositions, encoding interpersonal meanings such as obligation (e.g., *You must leave now*) and inclination (e.g., *I will help you*). While modalization positions speakers with respect to the truth-value or likelihood of a proposition, modulation operates in the domain of proposals, regulating how actions are to be taken up, resisted, or negotiated between interlocutors.

Within this system, **obligation modulation** refers to linguistic resources for expressing necessity, duty, and social expectation, typically realized through modal verbs or constructions such as *must*, *should*, or *be supposed to*. These resources allow speakers to propose actions and simultaneously express interpersonal judgments about their necessity or desirability. Through modulation of obligation, speakers manage interpersonal relations by invoking social expectations, negotiating authority, and proposing socially guided courses of action.

The cognitive operations underlying obligation modulation include the ability to model shared norms, to project social duties onto others, and to abstract behavioral expectations beyond immediate physical circumstances. In this way, obligation expressions function not merely to represent the world but to intervene in it by linguistically constructing socially sanctioned imperatives and directing interpersonal behavior. The cognitive underpinnings of this reduction are explored in the following discussion.

**(i) Predictive processing anomalies and social negotiation**

Research on ASD communication shows consistent pragmatic challenges, especially in managing socially contingent aspects of interaction, including topic maintenance, interpretation of conversational cues, and reference to mental states (Tager-Flusberg, 1993; Capps et al., 1998; Paul et al., 2009). Narratives by individuals with ASD often contain less evaluative content and fewer references to emotions or mental states (Capps et al., 1998), alongside difficulties in conversational reciprocity and responsiveness to listener needs (Paul et al., 2009). These patterns suggest a reduced engagement with language functions involving social positioning and subjective evaluation.

Within the SFL framework, such tendencies can be understood as diminished use of interpersonal resources—modulation systems like obligation presuppose the negotiation of social roles through language. A cognitive-linguistic preference for literal, concrete, and perceptually grounded meanings over socially constructed or abstract imperatives (Happé, 1997; Norbury, 2005; Martin and McDonald, 2003) may help explain the reduced naturalness of obligation modulation in ASD. From a predictive processing perspective, successful use of obligation forms requires anticipating interlocutors’ expectations and likely responses; individuals with ASD often show reduced sensitivity to such contingencies, leading to reduced use of modulation as a resource for social negotiation.

**(ii) Local bias and challenges in normative framing**

WCC theory proposes that individuals with ASD exhibit a cognitive style biased toward local, detail-focused processing at the expense of integrating information into global, coherent patterns (Frith, 1989; Happé and Frith, 2006). This bias manifests across perceptual, cognitive, and linguistic domains, leading to a preference for discrete, veridical information over higher-level abstractions.

Obligation expressions — such as *You must apologize* — require abstraction beyond the immediate situation. The utterance does not merely describe a present event but invokes a generalized social rule that extends across contexts. Such abstraction depends on integrating multiple situational cues, recognizing socially sanctioned patterns, and projecting these patterns onto the present interaction. For individuals with ASD, whose processing favors the immediate and concrete, this kind of normative abstraction is less spontaneously generated. Communication thus tends to remain anchored in observable particulars, rather than embedded in generalized frameworks of obligation.

However, local bias alone does not fully explain the reduced use of deontic expressions. A further factor is a deficit in social-pragmatic integration: the capacity to track shared knowledge, evaluate context-sensitive norms, and construct socially situated meanings in real time. This manifests in difficulties with emotionally grounded causal reasoning and context-dependent inference, especially when shared beliefs or intentions must be inferred from minimal input. Rather than drawing on higher-level schemas to guide interpretation, individuals with ASD often rely on immediate sensory details, leading to pragmatic flattening. This constrains their ability to encode social expectations and to acquire the linguistic routines necessary for dynamically adjusting obligation modulation in interaction.

In sum, obligation modulation in SFL is an interpersonal resource for negotiating duties and expectations. Its reduced use in ASD reflects limited engagement with social negotiation, stemming from difficulties in constructing shared frameworks, reduced orientation toward relational modulation, and a preference for factual description over socially guided imperatives.

### 4.6 Evaluative language

#### 4.6.1 Theoretical framework of appraisal

Evaluative language expresses feelings or judgments about the self, others, events, and objects from the speaker’s or writer’s perspective. Thompson and Hunston (2000, 6) identify three key functions: expressing opinions that reflect individual and community values, managing relationships between interlocutors, and organizing discourse. Evaluation operates at multiple linguistic levels—phonological, lexical, syntactic, and discursive. Within SFL, Martin and White (2005) developed appraisal theory to systematize this interpersonal dimension, expanding the metafunction to encompass resources for managing subjective and intersubjective stance. Appraisal consists of three domains: **Attitude** (resources for encoding emotions, assessments of behavior, and evaluations of value), **Graduation** (scaling of intensity), and **Engagement** (the speaker’s stance toward the proposition). The annotation scheme of Kato et al. (2022) incorporates attitude and graduation, allowing analysis of evaluative types and scalar variation.

##### 4.6.1.1 Attitude: categories and subtypes

Martin and White (2005) divide *attitude* into three domains: **Affect**, **Judgment**, and **Appreciation**. Affect encodes emotional responses (e.g., fear, sadness, happiness); Judgment evaluates moral or ethical behavior (e.g., brave, deceptive); and Appreciation assesses the esthetic or value-oriented qualities of entities and processes (e.g., elegant, innovative). Each domain has subcategories, with modifications introduced in the corpus of Kato et al. (2022).

**(i) Affect**

Affect encodes how speakers represent their emotional states—how they feel about something, rather than how it shapes their thoughts. It answers the question: *how did/do you feel about it?* Although typically realized through adjectives, affect can also appear incongruently as nouns, adverbs, or verbs (e.g., *comfort, cheerfully, fear, enjoy*). Following Martin and White (2005), but with modifications in Kato et al. (2022), *inclination* was added as a new category, and *happiness* was relabeled as *emotion*.

(1) **Inclination**: lexis encoding likes and dislikes toward things or persons (e.g., *like, favorable, dislike, hateful*).
(2) **Emotion**: lexis encoding sadness, anger, happiness, or love (e.g., *sad, distraught, happy, cheerful*).
(3) **Security**: lexis encoding anxiety or confidence (e.g., *anxious, uneasy, confident, composed*).
(4) **Satisfaction**: lexis encoding interest or disinterest (e.g., *absorbed, satisfied, dissatisfied*).

**(ii) Judgment**

Judgment refers to evaluative expressions relating to ethics, morality, and social values. It represents the speaker’s assessment of others’ verbal, mental, or physical behavior in terms of whether such behavior conforms to or diverges from normative standards. Judgmental evaluation addresses the question: *how would you judge that behavior?* Two major categories are distinguished—**social sanction** and **social esteem**—each comprising multiple subcategories. Following Martin and White (2005), but with modifications in Kato et al. (2022), the categories of **propensity** and **dependability** were introduced in place of *normality* and *tenacity* to better capture evaluative distinctions observed in the data.

(1) **Social sanction**: judgment focused on moral regulation, ethics, truthfulness, and conceptions of right and wrong. It consists of two subcategories:

(a) **Veracity**: lexis evaluating a person’s honesty or credibility (e.g., *honest, credible, trustworthy, frank, deceitful, dishonest, unconvincing, inconsistent, hypocritical*).
(b) **Propriety**: lexis assessing a person’s ethical or moral conduct, either as alignment with or violation of social norms (e.g., *moral, righteous, ethical, immoral, wrong, abusive*).

(2) **Social esteem**: evaluation of how well individuals meet socially valued standards of behavior. It consists of three subcategories:

(a) **Propensity**: lexis approving or disapproving of an individual’s behavioral tendencies or dispositions (e.g., *self-reliant, energetic, brave, cowardly*).
(b) **Capacity**: lexis judging a person’s ability or competence in performing actions (e.g., *skillful, incompetent, stupid, clever*).
(c) **Dependability**: lexis evaluating an individual’s reliability or consistency (e.g., *credible, truthful, staunch, upright, deceitful, fraudulent*).

**(iii) Appreciation**

*Appreciation* refers to evaluations of phenomena or entities based on esthetic or socially recognized value systems. Whereas *judgment* assesses human behavior, *appreciation* typically applies to tangible objects (e.g., artifacts, products) or abstract constructs, and when directed at people, treats them as perceptual entities rather than agents. Following Martin and White (2005), with modifications in Kato et al. (2022), the subcategory **social evaluation** was added to capture socially grounded assessments (e.g., credibility, utility), and **scalarity** was introduced to represent evaluative meanings related to extent, degree, and spatio-temporal dimensions.

(1) **Reaction**: evaluation based on emotional response (e.g., *interesting, boring, disappointing, inspiring*).
(2) **Composition**: evaluation of structure, organization, or balance (e.g., *well-balanced, complex, simple*).
(3) **Social evaluation**: assessment grounded in social conventions such as significance, credibility, or utility (e.g., *important, credible, effective, meaningless*).
(4) **Scalarity**: evaluation along time (e.g., *prolonged, brief*), extent (e.g., *widespread, limited*), degree (e.g., *intense, mild*), space (e.g., *spacious, cramped*), distance (e.g., *remote, close*), mass (e.g., *massive, slight*).

Five lexicogrammatical features—Judgment–veracity, Judgment–propriety, Affect–satisfaction, Appreciation–reaction, and Appreciation–scalarity-space—were found to occur less frequently in ASD discourse.

##### 4.6.1.2 The concept of affordances and its implications for appraisal

Individuals with ASD use judgmental evaluative expressions of veracity and propriety less frequently than non-ASD individuals. To interpret these differences, the concept of *affordances* provides a coherent framework, clarifying how limitations in social-cognitive processing shape evaluative expression within ecological and phenomenological contexts.

The verbal expression of *Judgment* depends on sensitivity to both institutional and interpersonal affordances. Reduced use of judgmental lexis in ASD can thus be understood as diminished responsiveness to these structures, which require attention to environmental, social, and normative cues simultaneously.

From a phenomenological standpoint, Merleau-Ponty (2013) emphasized that consciousness is inherently relational, grounded in *corporeity*—the embodied awareness that situates perception in context. Building on this view, Gibson (1979) introduced the concept of *affordances* to describe the actionable possibilities offered by the environment relative to the perceiver’s embodied capacities. Affordances are not intrinsic properties of objects but arise through engagement: a chair affords sitting, an apple affords eating, air affords breathing. Perceiving an affordance thus involves awareness of both the environment and one’s own agency, what Gibson terms the *ecological self*.

Affordances extend beyond sensorimotor interaction. Through social learning, objects acquire *intentional affordances* (Tomasello, 1999), such as a telephone affording communication rather than mere physical handling. Participation in symbolic practices further gives rise to *institutional affordances* (Fiebich, 2014), as when pruning shears, in the context of rock-paper-scissors, afford the gesture for “scissors.” Affordances therefore operate at multiple levels—sensorimotor, intentional, institutional—each requiring distinct forms of interpretive competence. Fiebich also distinguishes between narrow social cognition (understanding individual intentions) and broad social cognition (grasping collective, institutional intentions).

**Figure 2:**
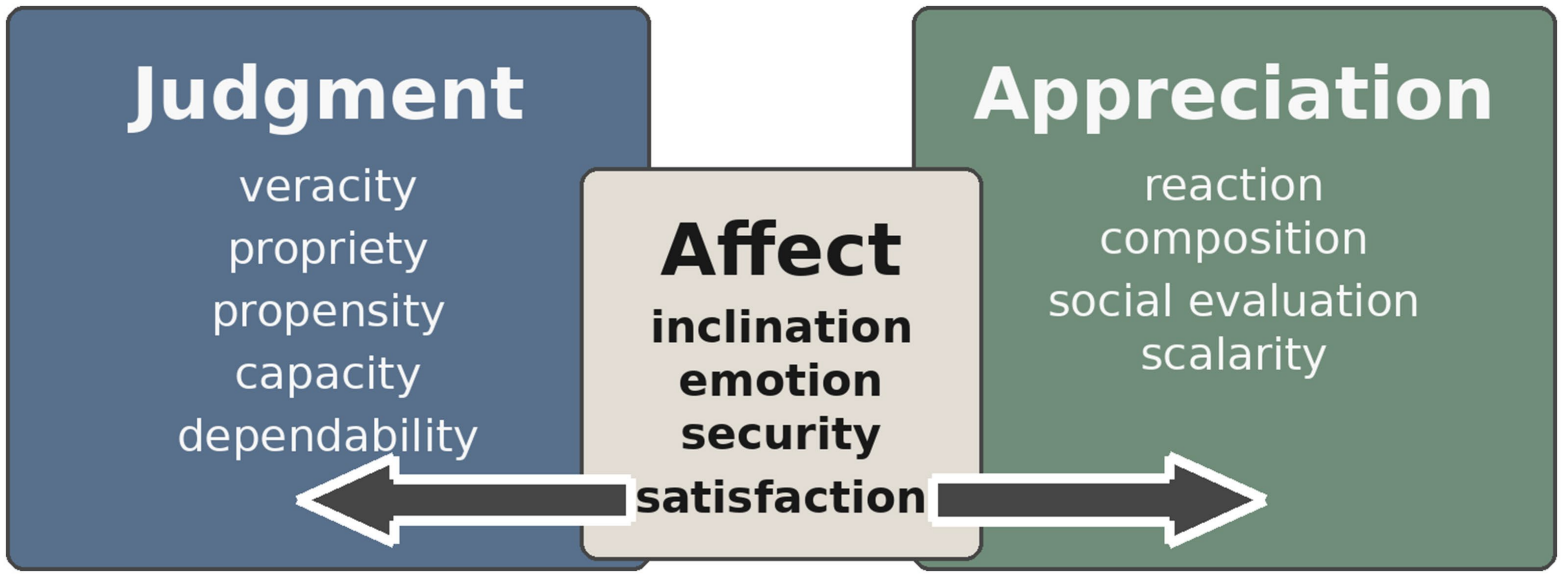
The three main categories of appraisal (adapted from Kato, 2023).

Within Appraisal theory, *Judgment* and *Appreciation* are conceptualized as institutionalized forms of affect (Martin and White, 2005; Figure 2): *Judgment* as moral/ethical evaluation of behavior, and *Appreciation* as value-oriented evaluation of entities and processes. To evaluate behavior as right or wrong presupposes not only personal stance but also the internalization of shared norms and sensitivity to institutional affordances. The reduced frequency of judgmental evaluation in ASD may therefore reflect limitations in perceiving and internalizing such affordances, linked to atypicalities in social cognition and normative engagement.

Affordances can broadly be divided into *ecological* (emerging from interaction with the physical environment) and *interpersonal* (emerging from interaction with others). Interpersonal affordances are a subset of ecological ones, dynamically shaped through mutual responsiveness (Fiebich, 2014; Honda, 2013). Because judgmental evaluation is especially sensitive to these interpersonal affordances, its reduced use in ASD underscores difficulties in aligning evaluative language with socially shared standards.

##### 4.6.1.3 The major factors impairing social learning

The capacity to perceive ecological affordances develops through ontogeny, shaped by maturation and social learning (Fiebich, 2014). The marked reduction of judgment-related lexis in ASD suggests underacquisition of institutional knowledge, limiting the development of socially relevant cognition. Perceiving institutional affordances requires sustained social learning, which depends on neurocognitive development. In ASD, this process is disrupted, with two factors most central: impairments in JA and a cognitive style marked by WCC.

**(i) JA deficits and their impact on social learning**

JA is the capacity to coordinate and share attentional focus, defined as “the awareness that oneself and another agent are attentive toward the same ecological entity and that this awareness is mutually shared” (Fiebich, 2014:154). It underlies interpersonal interaction and communication (Mundy et al., 1990) and is frequently impaired in ASD, where gaze-following, pointing, and object-sharing are reduced. Such deficits reflect not only cognitive but also affective impairments in intersubjectivity (Leekam, 2005).

Children with ASD often fail to orient to social cues such as eye gaze or name calls, despite responding reflexively to nonsocial stimuli. JA is also tied to social motivation (Mundy and Sigman, 2006): children who naturally attend to faces and voices acquire communicative skills through participation (Schietecatte et al., 2012). In contrast, ASD is characterized by reduced preference for social stimuli, atypical facial processing (Dawson et al., 2004; Sasson et al., 2007), and diminished orientation to faces (Sasson et al., 2007). These atypicalities constrain JA development and thereby limit opportunities for social learning and the acquisition of communicative and social knowledge.

**(ii) WCC and social information processing**

Within affordance perception, the cognitive profile of WCC helps explain the reduced use of judgmental evaluative expressions in ASD. A detail-focused processing style limits the construction of coherent representations of socially situated events, impeding recognition of institutional and interpersonal affordances distributed across multiple cues. Because Judgment presupposes sensitivity to implicit relational dynamics and shared norms, this local bias constrains the integration of social meaning and weakens alignment with collective evaluative frameworks. In this way, WCC reduces the capacity for evaluative positioning within socially governed contexts.

Together with JA deficits, WCC restricts participation in social interaction and deprives individuals with ASD of opportunities to acquire social rules and institutional knowledge through experience.

##### 4.6.1.4 Reduced use of satisfaction expressions in ASD discourse

The reduced use of satisfaction expressions in ASD cannot be attributed to a general emotional deficit, as no comparable reduction appears in inclination, emotion, or security. Unlike these more immediate emotional reactions, satisfaction requires integrating experiences into a holistic evaluation of fulfillment across time and context. Because individuals with ASD often show WCC, they may struggle to construct or verbalize such global summaries, leading to less frequent use of satisfaction-related expressions.

##### 4.6.1.5 Reduced reaction evaluations and scalarity-space

Individuals with ASD also show reduced use of evaluative expressions in the domains of reaction and scalarity-space within Appreciation. Reaction evaluates the salience or impact of phenomena (e.g., vivid, dull, impressive), while scalarity-space involves holistic assessments of spatial properties (e.g., spaciousness, openness). Reduced Reaction lexis indicates a focus on discrete details over global experiential impact, leading to descriptions that prioritize concrete information rather than evaluative construals of salience or vividness. Likewise, the reduction of scalarity-space lexis reflects WCC-related impairments in global spatial cognition: ASD speakers tend to process input locally, limiting their ability to perceive and verbalize overall spatial qualities.

These findings suggest that ASD-related differences in evaluative language extend beyond social and affective domains to encompass broader limitations in holistic processing and experiential evaluation.

#### 4.6.2 Graduation: categories and subtypes

Graduation, alongside Attitude and Engagement, is one of the three major subsystems of the Appraisal framework. It concerns the *scaling of meaning*—how speakers adjust the intensity or preciseness of evaluation (Martin and White, 2005). Graduation applies to both attitudinal and engagement resources and operates along two axes: Force and Focus.

**(i) Force**

Force refers to the up- or down-scaling of intensity, quantity, or extent, realized through two subtypes:

(1) **Intensification**: scaling qualities (e.g., *very* angry*, quite* good) or processes (e.g., *greatly* improved*, barely* noticed).
(2) **Quantification**: scaling number, mass, or frequency (e.g., *many, entirely, often*).

**(ii) Focus**

Focus concerns prototypicality—how closely an entity aligns with the core of a category. It modifies category boundaries by:

(1) **Sharpening** (up-scaling): *a real* friend*, a true* father.
(2) **Softening** (down-scaling): *kind of* strange, an apolog*y of sorts, sort of* upset.

Focus typically applies to non-gradable categories (e.g., *jazz music*). For example, *They play jazz, **sort of*** implies borderline membership in the category.

Graduation thus not only modulates the strength of evaluative meaning but also performs a dialogic role, expressing stance and calibrating interpersonal alignment. Within our analysis, two lexicogrammatical features emerged as discriminators: Force: intensification and quantification.

##### 4.6.2.1 Reduced use of force in ASD

The present findings indicates that individuals with ASD tend to use Graduation resources of Force—intensifiers and certain quantifiers—less frequently and with less flexibility than non-ASD speakers. In practice, this means fewer markers such as *very*, *extremely*, *completely*, and less frequent use of subjective or hyperbolic quantifiers. Autistic children often produce narratives with reduced evaluative richness and weaker coherence, reflecting difficulties in linking events and conveying their significance (Losh and Capps, 2003). This points to a difference not in vocabulary or grammar, but in the use of language for graduation resources of force. The reduced use of Force can be traced to several underlying cognitive constraints, outlined as follows:

**(i) WCC and local focus**

Individuals with ASD often excel at perceiving and recalling details but show difficulty integrating information into a coherent whole. Linguistically, this produces discourse that is concrete, literal, and fact-oriented, with less emphasis on global evaluative shading. Intensifiers and quantifiers often presuppose a judgment of overall significance—for example, whether an event was ***hugely** important* or ***barely** relevant*. If attention remains fixed on isolated details, there may be little motivation to scale meaning globally. Autistic narratives often describe sequential facts or actions without signaling their relative intensity, consistent with WCC accounts of local bias (Barnes and Baron-Cohen, 2010).

**(ii) Reduced global integration across context**

Related to WCC, autistic individuals frequently show difficulty integrating information across broader contexts. Force markers such as ***absolutely** essential* or ***extremely** rare* function to situate information within a wider evaluative frame, indicating its weight relative to other elements. If global coherence is not spontaneously constructed, speakers may default to reporting facts without adding such gradational cues. Their discourse can thus appear *flat*—not due to absence of emotion, but due to a communication style that prioritizes literal accuracy and uniform description.

**(iii) Pragmatic and social-communicative differences**

ASD is also associated with reduced sensitivity to pragmatic demands of interaction, including adapting language to signal stance, urgency, or interpersonal alignment. Intensifiers and quantifiers often serve dialogic purposes, calibrating how strongly a speaker positions themselves relative to the listener. If social interaction is experienced less as a shared field of meaning and more as a unilateral act of reporting, then the incentive to scale expressions for interpersonal effect is weakened. This leads to a more evenly modulated, minimally gradated style of expression, reflecting a difference in pragmatic orientation rather than a linguistic deficit.

**(iv) Literal interpretation and avoidance of figurative usage**

Many intensifiers and quantifiers are not literal measures but pragmatic exaggerations (*I’ve told you a million times*). Because individuals with ASD often show an over-literal orientation (Petit et al., 2025), they may avoid or fail to acquire such non-literal forms. Even a common intensifier like *literally* in *I literally died laughing* is figurative in function, and may be dispreferred by autistic speakers who prioritize semantic accuracy. The result is a discourse style that favors precise, literal terms (*I told you repeatedly*) over hyperbolic or figurative gradation.

Taken together, these accounts suggest that the reduced use of force markers in ASD arises from an interplay of cognitive and pragmatic traits: detail-focused processing, reduced global integration, attenuated intersubjective motivation, and literal semantic preference. Consequently, ASD discourse often appears structurally intact but affectively and interpersonally flatter, with less frequent use of gradational terms (*very, extremely, a lot, only a bit*) that, in non-ASD communication, help calibrate stance and foster alignment with interlocutors.

In sum, this section showed that ASD individuals make limited use of Attitude and Graduation resources. Attitude is constrained by reduced sensitivity to interpersonal and institutional affordances, shaped by JA and WCC. Graduation: Force, intensifiers and quantifiers, is underused, reflecting both cognitive style and diminished intersubjective negotiation.

### 4.7 Onomatopoeia as a discriminator: mimetic vs. imitative forms

Onomatopoeia is significantly less frequent in ASD discourse. Its use is not mere sound imitation but requires abstraction, categorization, and social conventionalization. The reduction was concentrated in mimetic onomatopoeia, whereas imitative forms showed no reliable difference, indicating that ASD-related difficulties emerge most in forms demanding cross-modal and pragmatic sensitivity.

#### 4.7.1 Abstraction and conventionalization demands in onomatopoeia

Using onomatopoeic words such as *splash*, *clang*, or *buzz* requires generalizing across varied auditory experiences, selecting salient features while ignoring others. Mimetic forms like *pika-pika* (glittering) or *shīn* (silence) demand even greater cross-modal abstraction from visual, tactile, or affective impressions. Such compression stabilizes sensory variability into culturally recognizable forms. In ASD, predictive processing atypicalities disrupt this process: with reduced reliance on prior expectations and greater dependence on bottom-up input (Pellicano and Burr, 2012), individuals show heightened sensitivity to fine-grained detail, making abstraction—especially for mimetic expressions—less intuitive.

#### 4.7.2 Predictive processing anomalies and the instability of sound-symbol mappings

Onomatopoeia depends on balancing prediction and perception: speakers anticipate that *bang* will evoke a sudden noise within a shared cultural frame. While imitative forms remain stable through recurring auditory events, mimetic forms require projecting non-auditory qualities (e.g., brightness, speed, stillness) onto sound, relying more heavily on prior expectations and cultural conventionalization. In ASD, reduced reliance on priors undermines the predictability of such mappings, making conventionalized sound-symbols less natural. Instead of invoking general terms like *crash*, individuals may focus on idiosyncratic details, treating each event as event-specific.

**(i) Weaknesses in global perceptual abstraction: effects of WCC**

Onomatopoeia requires filtering perceptual detail to foreground a salient sound profile. For imitative forms, this acoustic gestalt often remains accessible. Mimetic forms, however, demand cross-modal projection (e.g., mapping stillness or glitter into sound), which depends on global integration. Under WCC, attention remains on fine-grained differences, making it difficult to collapse heterogeneous experiences under a single conventional label.

**(ii) Executive function constraints on flexible abstraction and inhibition**

Limitations in cognitive flexibility and inhibitory control (Miyake et al., 2000; Hill, 2004; Ozonoff et al., 1991) hinder the use of onomatopoeia. Mimetic forms especially demand suppressing detailed sensory memories and choosing among many near-synonymous cultural variants (e.g., *pika-pika, gira-gira, kira-kira*). Imitative forms, more tightly tied to concrete sounds, impose a lighter executive load.

**(iii) Pragmatic motivational differences in communicative orientation**

Onomatopoeia often enhances vividness and emotional resonance, presupposing orientation toward the listener’s experience. Mimetic forms are listener-oriented, evoking mood or atmosphere. In ASD, reduced focus on the interlocutor’s affective perspective lowers their frequency, while imitative forms align better with factual description.

**(iv) Conventional arbitrariness and challenges in acquisition**

Onomatopoeia requires learning culturally specific, often arbitrary sound-meaning mappings, as in English *woof* versus Japanese *wan-wan* (both denoting a dog’s bark). For individuals with ASD, who prefer systematic rules, this arbitrariness presents added difficulty. The challenge is greatest in mimetic forms, which depend on cultural conventions rather than direct auditory anchors, whereas imitative forms remain more transparent.

#### 4.7.3 Integrative perspective

Predictive processing anomalies, local-detail biases, executive limitations, and pragmatic orientations jointly constrain the natural use of onomatopoeia in ASD. Producing such forms requires abstraction, inhibition, prediction, and cultural conventionalization—processes systematically affected in ASD. These constraints weigh most heavily on mimetic onomatopoeia, which demand cross-modal abstraction and pragmatic sensitivity, while imitative forms tied to direct auditory reproduction show no reliable group difference. This selective reduction understores how ASD-related challenges emerge most strongly in culturally conventionalized, higher-order sound-symbolic expressions.

In sum, the reduced use of onomatopoeia in ASD reflects constraints in predictive processing, global integration, and executive functions—especially cognitive flexibility and inhibitory control—together with pragmatic orientation. These demands—abstraction, conventionalization, and context-sensitive interpretation—pose marked difficulty in mimetic forms, while imitative forms tied to direct sound reproduction show no reliable group difference.

### 4.8 Limitations and future perspectives

The principal limitation of this study concerns the restricted sample size, which reduces the statistical robustness of the identified lexicogrammatical discriminators. Subsequent research incorporating a larger participant pool will permit more rigorous validation of the results and enhance the reliability of the cognitive interpretations derived from them.

Among the 46 statistically significant features identified in this study, several were not subjected to detailed interpretation. Their exclusion reflected empirical limitations, interpretive ambiguity, or redundancy with more tractable items. Constructions involving explanatory mood (*noda*) combined with negotiating particles were omitted, as *noda* itself did not emerge as an independent discriminator, while the negotiating particles have been analyzed in Kato et al. (2022) and Kato and Hanawa (2025). Filler expressions (e.g., *unto*, *kono*) likewise met statistical thresholds but were judged to reflect speaker-specific or interactional habits rather than systematic cognitive-functional patterns. These exclusions reflect a conservative stance prioritizing clarity, replicability, and theoretical grounding, while indicating areas where future research with multimodal or expanded corpora may yield further insights into ASD-related language use.

## 5 Conclusion

The findings of this study deepen our understanding of how choice patterns of lexicogrammar reflect domain-specific cognitive processing differences across the autism spectrum. The degree to which these discriminators appear varies with an individual’s position on the spectrum and with their verbal abilities, shaping how prominently particular features emerge in discourse. Because these features reliably distinguish ASD from non-ASD discourse, they also demonstrate diagnostic potential, offering complementary indicators to traditional assessment tools. By linking linguistic discriminators to cognitive-functional domains, the present analysis provides mechanistic insights for language intervention, identifying which structures may be reinforced and which may require compensatory support in language development. This approach allows interventions to be tailored to the heterogeneous needs of individuals across the spectrum, ranging from minimally verbal to highly verbal speakers.

At the same time, it is essential to recognize the role of language-specific lexicogrammatical and pragmatic systems. ASD-related linguistic features are not uniform across languages: some discriminators are typologically specific, as illustrated by Japanese negotiating particles without direct counterparts in English. Cross-linguistic studies are therefore necessary to establish typological patterns in ASD-related language use. The development of multilingual corpora will be crucial for clarifying how ASD-related lexicogrammatical tendencies manifest across different linguistic systems, contributing to a more global account of neurocognitive variation.

Finally, advances in computational linguistics, including large language models trained on diverse corpora, provide new opportunities to test and extend these findings. By integrating corpus-based analysis with language model simulations, it becomes possible to examine how lexicogrammatical features contribute to classification, to explore cross-linguistic generalizability, and to model the probabilistic tendencies that underlie ASD-related discourse. Such integration offers a powerful means of connecting cognitive theory, linguistic description, and computational modeling in future research.

## Data Availability

The raw data supporting the conclusions of this article will be made available by the authors, without undue reservation.
