# Supplementary material for: Lexicogrammatical profiling of ASD: cognitive-functional mapping and diagnostic implications
Source: Front Hum Neurosci. 2026 Jan 29;19:1704950. doi: 10.3389/fnhum.2025.1704950 (PMC12894288; doi:10.3389/fnhum.2025.1704950)
Supplement: Supplementary file 1 [file Data_Sheet_1.DOCX]

**Supplementary Material**

**Table S1**. Overview of Demographic and Clinical Metrics in ASD vs. Non-ASD Populations.

| Characteristic | ASD Group | Non-ASD Group | P-Value |
| --- | --- | --- | --- |
|  | (N=64) | (N=71) |  |
| Age (years) | 18 ± 3.48 | 19 ± 2.77 | 0 |
| Sex (M/F) | 24/40 | 39/32 | 0.06 |
| Education | N/A | College GPA range: 2.4 - 2.8 | N/A |
| ADOS-2 Module 3 | 6.93 ± 1.38 | 2.75 ± 2.01 | < 0.01 |
| ADOS-2 Module 4 | 11.42 ± 3.55 | 4.22 ± 2.17 | < 0.01 |
| SRS-2 Total Score | 85.53 ± 9.00 | N/A | N/A |
| WISC-IV-IQ Full Scale IQ | 81.22 ± 14.42 | N/A | N/A |
| WAIS-III Full Scale IQ | 91.33 ± 20.12 | N/A | N/A |
| Vineland-II Composite | 64.83 ± 22.53 | N/A | N/A |
| AQ | 36.64 ± 8.04 | N/A | N/A |
| PARS-TR Preschoolers | 13.54 ± 6.27 | N/A | N/A |
| PARS-TR Adolescents & adults | 24.30 ± 11.49 | N/A | N/A |

**Table S2.** Tag types and linguistic functions.

| **Lexicogrammar headings** | **Linguistic functions** | | **Tag types** | **No. of tag types** |
| --- | --- | --- | --- | --- |
| **Ideational metafunction** | | | | |
| 1. Process type | The mental image of reality is constructed by the TRANSITIVITY (clause component) of a clause. All individuals create a representation of reality. Experiential worlds are defined using 10 types of process verbs, yielding information about how, when speaking, an individual creates a representation of reality. | | 1.Material-doing 2.Material-happen 3.Mental-cognition 4.Mental-affect 5.Mental-perception 6.Relational-attribute 7.Relational-identity 8.Behavioral 9.Verbal 10.Existential | 10 |
| 2. Ergativity | This measures causation or instigation. In an ergative analysis, the participant that causes an event is the agent. Ergativity reveals whether a speaker interprets events and reality from the causal viewpoint of agency (effective) or becoming (i.e., a perspective lacking agency; a middle). | | 1.effective 2.middle | 2 |
| 3. Transitivity | A property that yields clues regarding the perspective (active or passive) from which the speaker interprets events and reality. | | voice (1.passive/active 2.causative) | 2 |
| 4. Clause complexes | The Japanese sentence type (of 22 types) chosen. This reveals syntactic ability and any cognitive tendency or deficiency. | | 1.Parallel clauses 2.*Te*-form/Conjunctive clauses-parallel/contrast 3.*Te*-form/Conjunctive clauses- forerunner 4.*Te*-form/Conjunctive clauses-sequence of actions 5.*Te*-form/Conjunctive clauses-cause/ reason 6.*Te*-form/Conjunctive clauses-adversative connective 7.*Te*-form/Conjunctive clauses-resultative condition 8.*Te*-form/Conjunctive clauses-attendant circumstance 9.Conditional clauses-resultative condition 10.Conditional clauses-converse condition-converse condition 11.Conditional clauses-converse condition-adversative connective 12.Conditional clauses-cause/ reason 13.Purpose clauses 14.Time clauses-temporal anteroposterior relation 15.Time clauses-simultaneous actions 16.Time clauses-others 17.Manner clauses 18.Reported clauses 19.Interrogative clauses 20.Noun clauses 21.Adnominal clauses 22.Cordinate clauses | 22 |
| 5. Logico- semantic relation | Logical clause linkages revealing syntactic ability, discourse strategy, and any cognitive tendency or deficiency. | | 1.Expansion-elaboration-expository 2.Expansion-elaboration-exemplifying 3.Expansion-elaboration-clarifying 4.Expansion-extension-additive 5.Expansion-extension-alternative 6.Expansion-enhancement-temporal 7.Expansion-enhancement-spatial 8.Expansion-enhancement-manner 9.Expansion-enhancement-cause-conditional 10.Projection-quote 11.Projection-report 12.Projection-idea 13.Projection-embedding | 13 |
| 6. Auxiliary verbs | **Stative:** Verbs describing the state of a subject rather than an action, reflecting the perspective of a speaker on an ongoing phenomenon. | | stative: (9 categories) compound: (1 category) | 10 |
|  | **Compound:** Verbs created by adding one verb to the stem of another; use of these verbs reflects the morphological skill of a speaker. | |  |  |
| **Interpersonal metafunction** | | | | |
| 7. Modality | In SFL, modality refers to an area of meaning that lies between yes and no; this constitutes the intermediate space between positive and negative polarity, categorized as either modalization (epistemic modality) and modulation. | | 1.Ability 2.Probability 3.Usuality 4.Necessity 5.Obligation 6.Permission 7.Expectation 8.Inclination 9.Modal Adjunct/Probability 10.Modal Adjunct/Usuality | 10 |
| 8. Appraisal- attitude | The semantic resource used to negotiate emotional reactions, judge behavior, and value things. Attitude is divided into three domains: affect, judgment, and appreciation. Affect is used to interpret emotional responses (including fear, loathing, sadness, and happiness); judgment is used for moral evaluation of behavior (including ethical, brave, and deceptive); and appreciation is used to interpret the esthetic qualities of semiotic phrases/processes and natural phenomena (including remarkable, desirable, elegant, harmonious, and innovative). This lexicogrammar reveals the speaker’s value system. | | 1.AFFECT-inclination 2.AFFECT-emotion 3.AFFECT- security 4.AFFECT-satisfaction 5.JUDGEMENT-capacity 6. JUDGEMENT-reliability 7.JUDGEMENT-veracity 8.JUDGEMENT-propriety 9.JUDGEMENT-propencity 10.APPRECIATION-reaction 11.APPRECIATION-composition 12.APPRECIATION-phase-time 13.APPRECIATION-phase-extent 14.APPRECIATION-phase-degree 15.APPRECIATION-phase-space 16.APPRECIATION-phase-distance 17.APPRECIATION-phase-mass 18.APPRECIATION-social evaluation | 18 |
| 9. Appraisal- graduation | This is one of the three categories that make up Appraisal, along with Appraisal-attitude, which focuses on gradability. (i.e., adjustment of the extent of evaluation). | | 1.FORCE-intensification 2.FORCE-quantification 3.FOCUS-sharpening 4.FOCUS-softening | 4 |
| 10. Negotiating particles | A lexis that adds various negotiatory values to a clause, implying the attitudinal stance of a speaker toward a proposition or proposal; this lexis is associated with a call for attention and indicates the territory of the information involved. | | sentence-final:1.*kana* 2.*kane* 3.*sa* 4.*ne* 5.*yo* 6.*yona* 7.*yone* Mid sentence:8.*kane* 9.*sa* 10.*ne* 11.*yo* Other:12.*sa* 13.*ne* | 13 |
| 11. Explanatory mood | An optional lexicogrammar often added to other mood types such as declarative and interrogative, implying a variety of meanings. It constitutes a cause, reason, motivation, source, and/or grounds for judgment that suggest a causal relationship between the explained and the explainer. | | 1.Explanatory mood 2.Explanatory mood:*ka* 3.Explanatory mood:*kana* 4.Explanatory mood:*kane* 5.Explanatory mood:*kedo* 6.Explanatory mood:other 7.Explanatory mood:*na* 8.Explanatory mood:*ne* 9.Explanatory mood:*yo* 10.Explanatory mood:*yone* 11.Explanatory mood:*yona* 12.Explanatory mood:*monoda* | 12 |
| 12. Evidentiality | This lexicogrammar describes how a speaker judges the validity of a proposition. Three types of evidence are used. *Appearance* refers to how the information is likely to appear or eventually occur; *hearsay* refers to how it will be known whether the event occurs; and *reasoning* refers to the reason the judgment is made or how the event is known to happen. | | 1.appearance 2.hearsay 3.reasoning | 3 |
| 13. Optative mood | A *desire* or *urge* to do something that the speaker considers desirable. | | lexis to express desire to do something | 1 |
| 14. Auxiliary verbs, benefactive | Verbs used when two parties converse; one party is doing something that benefits the other, and the other party is the recipient of that benefit. Such verbs indicate whether the speaker positions the other party inside or outside. | | Benefactive: (10 categories) | 10 |
| 15. Onomatopoeia | Imitative and mimetic words used to express manner, quality, or an exclamation. | | 1.imitative words 2.imitative mimetic words | 2 |
| 16. Filler | A time-filler: a meaningless sound, word, or phrase used in social settings when an individual is aware that a listener is present. | | Filler words-1.*maa* 2.*nanka* 3.*ano* 4.*unto* 5.*eeto* 6.*sono* 7.*kono* 8.*kou* | 8 |
|  |  |  | Total | 140 |

**Table S3.** Abbreviations used in interlinear glossing.

| Abbreviation | Full spelling | Notes |
| --- | --- | --- |
| ACC | accusative |  |
| APP | appearance | Use when emphasizing appearance (～*sō*) |
| BEN | benefactive | Use for ～*tekureru*, ～*temorau* etc. |
| COMP | complementizer | e.g., *to* in Japanese |
| COMPL | completive aspect |  |
| COND | conditional |  |
| CONJ | conjunction | Use when specific type is not required |
| COORD | coordinator | For and/or-type coordination particles like *ya*, *to* |
| COP | copula |  |
| DAT | dative |  |
| ETC | et cetera | Used for Japanese *toka*, *nado*, meaning “and so on”, “for example” |
| EVD | evidential | For hearsay, inference, appearance forms like ～*sō* |
| FP | final particle | Sentence-final discourse marker |
| GEN | genitive |  |
| GER | gerundive | Connective -te (clausal linker expressing cause/reason) |
| HEARSAY | hearsay evidential | Common glossing for the Japanese form 'sōda' when used as hearsay. |
| HON | honorific |  |
| INF | inferential | For internal judgments (e.g., ～*darō*) |
| LOC | locative |  |
| MOD | modal | For epistemic/deontic modals |
| NEG | negative |  |
| NEUT | neutral |  |
| NEX | non-exhaustive | For *ya, to* (non-exhaustive coordination/listing) |
| NMLZ | nominalizer |  |
| NOM | nominative |  |
| POL | polite | Appropriate for Japanese honorific forms |
| PROG | progressive |  |
| PST | past |  |
| Q | question particle |  |
| REC | recipient |  |
| REP | representative conjunctive | Used to gloss Japanese verbal ending “-tari”. |
| TE |  | Connective -te form |
| TOP | topic | Common in Japanese glossing |
| TRY | try | Trial auxiliary (-miru), expressing attempt or trying something out |


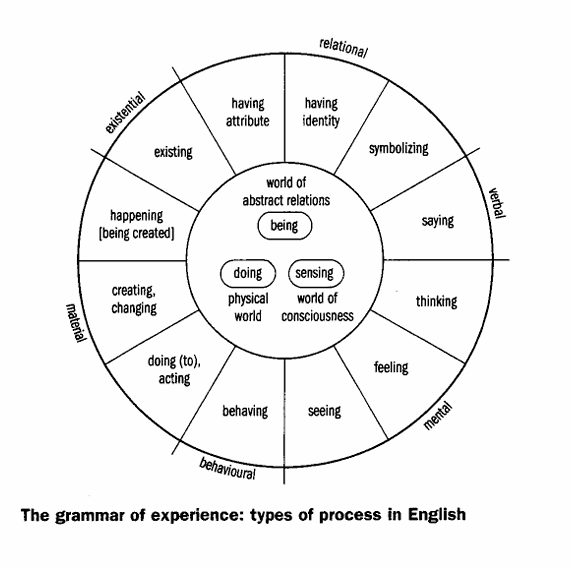


**Figure S1.** The grammar of experience: types of process in English.

**Material S1.** **Interview questions from Module 4 (Corsello et al., 2012)**

**Social Difficulties and Annoyance**

**FOR ADULTS (PRESENTLY NOT IN SCHOOL)**

- Do you have a job?

***If so:***

• What kind of job is it? How did you find it?

• Have you had other jobs before?

• Are you happy where you are or would you like to move on to something else eventually? What would it be?

• What about your co-workers? Do they seem to be happy or are they ready to move on?

***If not:***

• What do you do during the day?

• Did you have a job before? Why did you leave your old job? Was it something you had planned?

• Would you like to have a job someday?

***If yes:***

- What would it be?

- What will you need to do to find this type of job?

***If no:***

- What would you like to do?

**OR FOR STUDENTS (ABOVE JUNIOR HIGH SCHOOL AGES)**

Interview Questions About School

- Are you in school? Where?
- What courses are you taking?
- What year (grade) are you in? How is it going?

***If the participant is no longer in school and not employed:***

- How far did you go in school? How did it go?
- What do you plan to do next? What experience/training would you need to do that?
- Have you ever saved your money to buy something or do something special? What was it?
- Where are you living now?

***If living at home with parents:***

• Have you ever lived away from your parents?

• What would be different about living on your own?

• Would you like it better?

• What would be difficult?

***If living on his or her own:***

• How did you find the place where you live now?

• Who do you live with?

• Can you tell me a little about it?

- What do you like to do in your spare time at home?
- What about going out?
- What do you like doing that makes you feel happy and cheerful?
- What about things that you’re afraid of? What makes you feel frightened or anxious? How does it feel? What do you do?
- What about feeling angry? What kinds of things make you feel that way? How do you feel “inside” when you’re angry?
- Most people have times when they feel sad. What kinds of things make you feel that way?
- How do you feel when you’re sad? What is it like when you’re sad? Can you describe that?
- How about feeling relaxed or content? What kinds of things make you feel that way?
- Have you ever had problems getting along with people at school? How about at home with your family? Do you ever get in trouble? Why? What for?
- Are there things that other people do that irritate or annoy you? What are they?
- What about things you do that annoy others?-(if no response, ask: What about your brother(s) or sister(s) or parents?)
- Have you ever been teased or bullied? Why, do you think?
- Have you ever tried to change these things? Have you ever done anything so that others wouldn’t tease you? How has it worked?
- Are there other kids/people you know who get teased or bullied?
- Do you have some friends? Can you tell me about them?
- What do you like doing together? How did you get to know them? How often do you get together?
- What does being a friend mean to you? How do you know someone is your friend?
- How is a friend different from someone whom you just go to school with?
- Do you have a girlfriend or boyfriend? What is her/his name? How old is she/he?
- When did you see her/him last?
- What is she/he like? What do you like to do together?
- How do you know she/he is your girlfriend/boyfriend?
- Where do you want to live when you get older? What kind of place (apartment, house, condo)?
- Whom do you think you would like to live with? Your family, a roommate(s), by yourself?
- Do you ever think about having a long-term relationship or getting married (when you are older)?
- Why do you think some people get married or live with a girlfriend or boyfriend when they grow up?
- What would be nice about it? What might be difficult about being married or living with a girlfriend or boyfriend? Or living with a roommate?
- Do you ever feel lonely?
- Do you think other kids/people your age ever feel lonely?
- Are there things that you do to help yourself feel better? What about things other people do to help themselves feel better when they’re lonely?
